# Complex situations: Economic insecurity, mental health, and substance use among pregnant women who consider – but do not have – abortions

**DOI:** 10.1371/journal.pone.0226004

**Published:** 2020-01-15

**Authors:** Sarah C. M. Roberts, Nancy F. Berglas, Katrina Kimport

**Affiliations:** Advancing New Standards in Reproductive Health (ANSIRH), Bixby Center for Global Reproductive Health, Department of Obstetrics, Gynecology & Reproductive Sciences University of California San Francisco (UCSF), San Francisco, California, United States of America; Addis Ababa University School of Public Health, ETHIOPIA

## Abstract

We examine characteristics and experiences of women who considered, but did not have, an abortion for this pregnancy. Participants were recruited at prenatal care clinics in Louisiana and Maryland for a mixed-methods study (N = 589). On self-administered surveys and structured interviews, participants were asked if they had considered abortion for this pregnancy and, if so, reasons they did not obtain one. A subset (n = 83), including participants who considered abortion for this pregnancy, completed in-depth phone interviews. Multivariable logistic regression analyses examined characteristics associated with having considered abortion and experiencing a policy-related barrier to having an abortion; analyses focused on economic insecurity and of mental health/substance use as main predictors of interest. Louisiana interviews (n = 43) were analyzed using modified grounded theory to understand concrete experiences of policy-related factors. In regression analyses, women who reported greater economic insecurity (aOR 1.21 [95% CI 1.17, 1.26]) and more mental health diagnoses/substance use (aOR 1.29 [1.16, 1.45] had higher odds of having considered abortion. Those who reported greater economic insecurity (aOR 1.50 [1.09, 2.08]) and more mental health diagnoses/substance use (aOR 1.45 [95% CI 1.03, 2.05] had higher odds of reporting policy-related barriers. Interviewees who considered abortion and were subject to multiple restrictions on abortion identified material and instrumental impacts of policies that, collectively, contributed to them not having an abortion. Many described simultaneously navigating economic insecurity, mental health disorders, substance use, and interpersonal opposition to abortion from family and the man involved in the pregnancy. Current restrictive abortion policies appear to have more of an impact on women who report greater economic insecurity and more mental health diagnoses/substance use. These policies work in concert with each other, with people’s individual complex situations–including economic insecurity, mental health, and substance use–and with anti-abortion attitudes of other people to make abortion care impossible for some pregnant women to access.

## Introduction

In the first half of 2019, seven states have passed laws that ban abortions after 6-weeks of pregnancy, before many women know they are pregnant [1].These bans are a direct legal challenge to Roe v. Wade[2, 3] the U.S. Supreme Court decision that legalized abortion throughout the U.S. in 1973 [4].

What these bans purport to accomplish–making abortion impossible to obtain in that state–is already happening in some states for some women [5, 6]. There is a substantial and growing body of literature examining impacts of extant restrictive abortion policies and women’s experiences of these policies Previous research indicates some restrictive abortion policies–such as gestational limits, laws requiring abortion providers to have hospital admitting privileges (and which can lead abortion clinics to close), lack of Medicaid funding to pay for low-income women’s abortions, and multiple policies in combination–put abortion out of reach for some women [5–9]. There is also a robust body of research that documents adverse impacts on women and children when women are unable to obtain wanted abortions; these include: economic insecurity, adverse physical health impacts, ongoing violence from the man involved in the pregnancy, and adverse impacts on children’s development and maternal bonding [10–14].

More states now have multiple simultaneous restrictive policies in place [15]; thus previous research that examined impacts of restrictive abortion policies one-by-one may be less relevant for women’s actual experiences in current policy environments. A few studies have begun to examine impacts of and experiences with multiple simultaneous restrictive abortion policies. Two studies examined impacts of Texas’s HB2 law that had two restrictive policies go into effect at the same time, attributing a 13% reduction in the abortion rate due to the two policies, and found that the few women who did not obtain abortions described insufficient information about alternative open abortion facilities and lack of money and time as contributing to them continuing their pregnancies after the clinics closed [7, 16].

There has been less research, though, about which groups of women are affected by multiple simultaneous restrictive abortion policies and how their individual characteristics and policies interact to become barriers to abortion care. Understanding who is affected by policies and how policies affect them can help identify current disproportionate impacts of policies, inform efforts to mitigate harms of current policies, and inform public health plans for supporting women in possible future more restrictive environments.

In this manuscript, we examine characteristics of women who considered, but did not have, an abortion. Based on previous research that finds economic insecurity, mental health, and substance use contribute to delay in discovering pregnancy [17, 18] and in presenting for abortion care [19], we examine these factors in particular. We then explore ways policies concretely contribute to women not having abortions after considering them.

## Materials and methods

Data used in analyses presented in this manuscript come from the Louisiana and Maryland Abortion Prenatal Study. The Abortion Prenatal Study is a mixed-methods study of experiences related to considering and seeking abortion among women recruited at their first prenatal care visits in Southern Louisiana and Baltimore, Maryland [5]. A key original aim of the study was to study the impact of Louisiana’s Hospital Admitting Privileges law [20, 21], had that law gone into effect during the study time period. We also aimed to: 1) Understand women’s experiences considering and seeking abortions in a state with multiple versus few restrictive abortion policies; 2) Assess health & social service needs of women who consider but do not have abortions; and 3) Understand women’s experience with and impacts of Pregnancy Resource Centers. We have previously published some findings related to each aim [5, 8, 22–24]]. The analyses presented in this manuscript are a-priori components of the first aim. We have previously published findings that quantify differences in considering abortion and reporting a policy-related barrier to abortion between women living in a state with multiple versus few restrictive abortion policies [5], and quantify the impact of the policy-related barrier to abortion–lack of Medicaid coverage of abortion–that was most commonly referenced by study participants [8]. Here, we extend previous findings by 1) using multivariable analysis to rigorously assess individual-level characteristics of those who consider but do not have abortions and those who report policy-related barriers to abortion and 2) bringing in the qualitative component of the study to explore the concrete ways policies become barriers to abortion care.

### Study design

**Fig 1:**
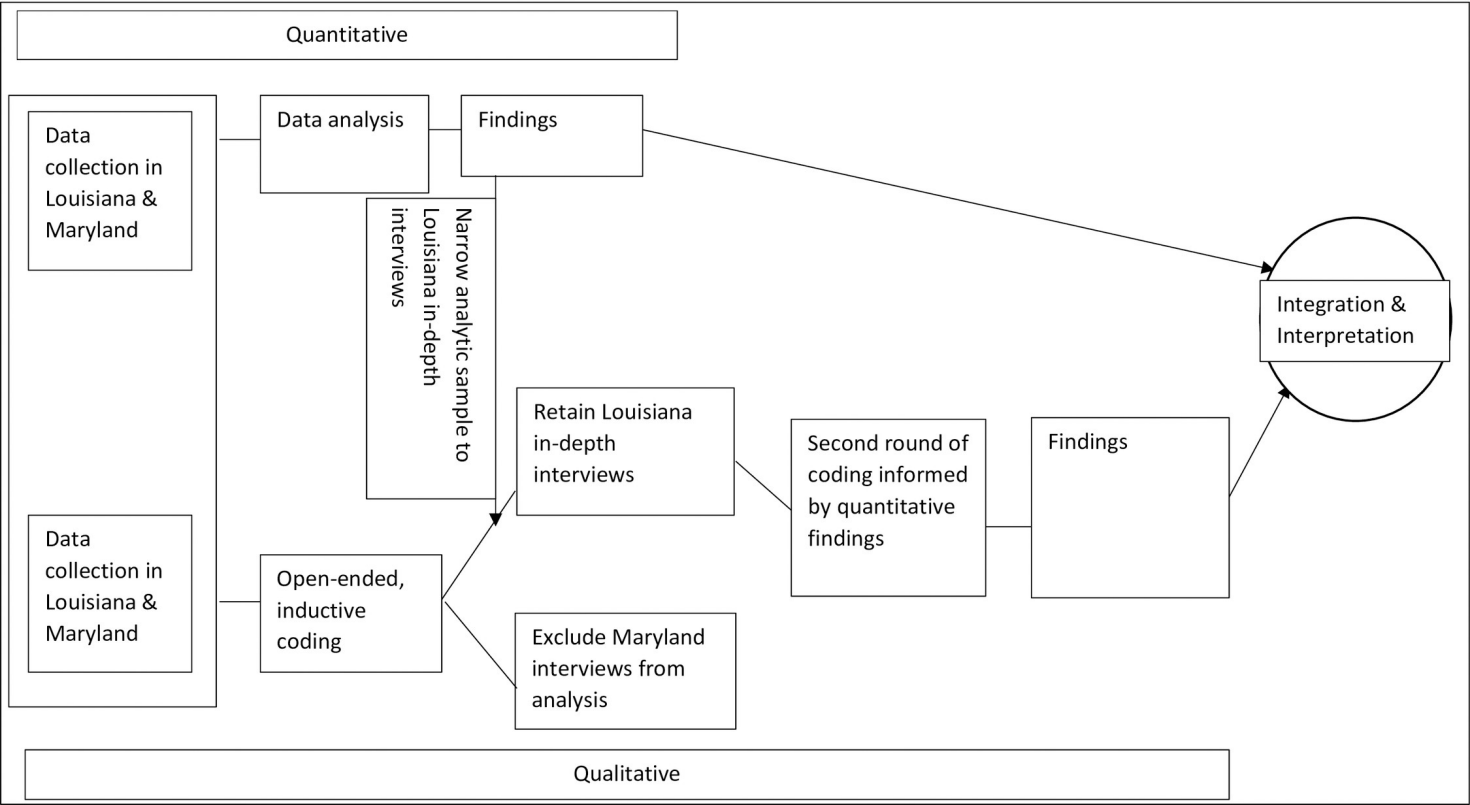
Relationship between quantitative and qualitative study components.

In this mixed-methods study, quantitative and qualitative data collections were convergent and embedded (Fig 1). Study methods have been described in detail previously [5, 8, 22, 23]. Briefly, we recruited 589 English and Spanish-speaking pregnant women 18 years and older at their first prenatal care visit at three prenatal care facilities in Southern Louisiana and one prenatal care facility in Baltimore, Maryland between June 2015 and June 2017. Study sites were university-affiliated prenatal care clinics that served primarily low-income pregnant women, many of whom were eligible for Medicaid insurance for prenatal care. At their first prenatal care visits, participants completed self-administered iPad surveys, followed by brief in-clinic structured interviews with a research coordinator. A purposively sampled subset (n = 83) completed an in-depth telephone interview one to four weeks later. The University of California, San Francisco Institutional Review Board and the Louisiana State University Health Sciences Campus Institutional Review Boards granted ethical approval for this study. The University of Maryland Institutional Review Board relied on the approval of the University of California, San Francisco Institutional Review Board.

### Study sites

Louisiana was selected because it has been classified as one of the most restrictive states in terms of abortion [25]. Louisiana also has multiple laws restricting abortion, including a parental involvement law for minors; mandatory waiting periods and ultrasounds; a 20-week ban; and lack of state-funding to pay for abortion for low-income women [26]. When the study launched, Louisiana had five abortion clinics [26]. By the end of recruitment, Louisiana had only three abortion clinics. To explore whether experiences considering and then not having abortions vary across states with different abortion policy and service availability environments, Baltimore, Maryland, was added as a comparison site because of its similarities to Southern Louisiana in terms of race/ethnicity, poverty, and birth rate, but very different abortion policy and service availability climate. Maryland has one law (a parental involvement law for minors) restricting abortion and uses state funds to pay for abortions for low-income women [27]. When the study launched in Baltimore, there were 25 abortion clinics in Maryland [28]; there were 24 at the end of recruitment [29].

### Study procedures

In each recruitment facility, an onsite research coordinator approached all women 18 and over who spoke English and were presenting for first prenatal care appointments at a participating site during the study time period. Partway through the first recruitment year, the research coordinator began approaching women who spoke Spanish. The research coordinator recruited all potentially eligible participants, screened them for eligibility, obtained written informed consent, and enrolled them in the study. After participants completed the self-administered survey, the research coordinator conducted a 5 to 15 minute in-clinic structured interview. Participants received $30 gift cards to remunerate them for in-clinic portions of the study.

In parallel, a purposively sampled subset of participants were invited to complete in-depth interviews by telephone. Interviews were designed to address a primary research question of how state abortion policies affect women’s ability to obtain an abortion. Initial criteria for being invited to complete an in-depth interview included reporting an unintended pregnancy, having considered abortion for this pregnancy, and/or having visited a pregnancy resource center. Over the course of recruitment, criteria were adjusted to ensure inclusion of participants with abortion-seeking experiences not yet represented in the sample. We ceased recruitment when the in-clinic portion of the study reached its pre-specified sample size. 83 respondents (43 Louisiana and 40 Maryland) completed in-depth interviews. Only women who spoke English were eligible for the in-depth interview, as the person conducting the in-depth interviews did not speak fluent Spanish.

Interviews were semi-structured, following a general interview guide but allowing respondents to introduce topics they found relevant. The interview guide (See S2 File. In-depth Interview Guide) covered several pregnancy- and abortion-related topics, including discovery of pregnancy, consideration of and attempts to obtain an abortion, use of other pregnancy-related service providers, experience of prenatal care, and thoughts on how easy or hard it is to obtain an abortion in their state. Consistent with feminist interviewing methods, the interviewer completed extensive field notes after each interview, summarizing content, identifying initial patterns, and reflexively accounting for her own social location and experience of the interview. Interviews averaged one hour in length; length did not differ by recruitment site. All interviews were audio-recorded with permission of participants and transcribed verbatim by a professional transcription company. Respondents were offered a $50 gift card to remunerate them for their time.

### Quantitative measures

On the self-administered survey and during in-clinic interviews, participants were asked if they had considered abortion at any point during their pregnancy “even for just one second.” During in-clinic interviews, those who had considered abortion were asked open-ended questions about the reasons they did not have an abortion. Responses were categorized using a-priori codes of personal reason, interpersonal reason, interaction with clinic or provider, and policy-related barrier. This coding process is described elsewhere [5]. Current analyses focus on two dichotomous outcomes: having considered abortion at any point during this pregnancy and reporting they did not have an abortion due to a policy-related barrier.

The self-administered survey also included variables related to participants’ backgrounds. Demographic variables included age (continuous) and race/ethnicity (categorical). SES variables included current unemployment, current lack of health insurance, use of public assistance in the last 12 months, housing insecurity in the last 12 months, and food insecurity in the last 12 months (all dichotomous). These variables were summed to create an economic insecurity index (range 0–5, with a higher score indicating a greater number of SES challenges faced by the participant). MH/SU variables included past 12-month alcohol use disorder risk (measured by the AUDIT-C scale [30] modified to capture binge drinking at > = 4 drinks at a time), any binge drinking, any illicit drug use including marijuana, and tobacco use as well as history of depression and history of anxiety (all dichotomous). These variables were summed to create a “mental health/substance use” index (range 0–6, with a higher score indicating more mental health history and more substance use). Participants also reported previous births (dichotomous), previous abortion (dichotomous), tobacco use, and previous involvement with Child Protective Services (CPS) (dichotomous). (See S1 File for survey and structured interview questions relevant to analyses presented in this manuscript).

### Analyses

#### Quantitative analyses

To examine predictors of each outcome variable (having considered abortion and reporting a policy-related barrier as a reason for not having an abortion), we first assessed bivariate relationships with participant characteristics using simple logistic regression models. We then ran two sets of multivariable logistic regression models for each outcome. The first set of models included all variables that were statistically significant at p<0.10 in bivariate analyses. The one exception was when binge drinking and alcohol use disorder risk were both significant in bivariate analyses; given the high correlation between these variables, we included one (alcohol use disorder risk) in multivariable models. The second set of models replaced dichotomous substance use and mental health variables with the two indices (economic insecurity and mental health/substance use), to understand if such challenges have additive effects. Third, as a sensitivity analysis to try to distinguish predictors of having considered abortion from predictors of experiencing policy-related barriers among those who considered abortion, we then replicated the policy-related barriers analysis, restricting the sample to those who had considered abortion. Fourth, because state was such a strong predictor of reporting a policy-related barrier as a reason for not obtaining an abortion [5] and thus including the Maryland sample in analyses of predictors of policy-related barriers might dilute results for the Louisiana sample (where the bulk of policy-related barriers were reported), we then replicated the policy-related barrier analyses among Louisiana participants only. All models used clustered standard errors (using Stata’s vce cluster command) to account for non-independent observations within recruitment facility.

#### Qualitative analyses

We analyzed the qualitative data to illuminate how restrictive abortion policies affect women’s ability to obtain an abortion. Because so few (n = 4 in the quantitative sample) of the Maryland participants reported a policy-related barrier[5] and our focus in the qualitative analysis was on how restrictive abortion policies affect the ability to obtain abortion care, we restricted analysis to in-depth interviews with women who were subject to multiple restrictive abortion policies, i.e. recruited in Louisiana (n = 43). Building off field notes and open-coding of the interview transcripts using techniques of modified grounded theory (Charmaz 2006), the senior author generated a preliminary code list. Initial codes were informed by the sensitizing concept (Charmaz 2006) of abortion-related decision points in respondents’ accounts and by the categories of the open-ended quantitative data coding. For this round of analysis, application of codes to transcripts was independent of coding of the in-clinic data. The senior author applied codes from this preliminary list to the transcripts in Atlas.ti software, with emergent codes added. Once all transcripts had been initially coded and no new codes capturing respondents’ decision-making process emerged, the code list was considered complete. The senior author then reviewed coding on all transcripts using the final code list.

After the quantitative analyses were complete and the importance of economic insecurity and mental health/substance use identified, the first and senior authors revisited the transcripts, particularly examining ways that these factors related to experiences with the restrictive abortion policies. Through discussion and memo-ing, the first and senior author identified relevant themes and patterns, resolving differences through discussion until we reached mutual agreement. In addition, throughout this process, the full team regularly discussed emergent findings, drawing on their familiarity with the data to evaluate the believability and trustworthiness of the findings. Names used below are pseudonyms.

## Results

### Quantitative findings

#### Quantitative sample description

86% of eligible participants in both states consented to participate (285/331 in Louisiana and 304/352 in Maryland). The sample includes the 586 women who initiated the iPad survey (99% of those who consented), among whom 95% (559) completed both the iPad survey and the in-clinic interview. Reporting having considered abortion in the iPad survey was not associated with completing the in-clinic interview.

Participants ranged in age from 18 to 44 years (mean 27.0, SD 5.6), and most were Black (79%) (Table 1). Most participants reported receiving public assistance in the past year (75%), and currently had public or private insurance (85%). About half were unemployed (49%) and reported food insecurity (47%) in the past year. On average, participants reported 2.1 (SD 1.2) SES challenges. A substantial minority reported binge drinking (36%), alcohol use disorder risk (26%), drug use (19%), and tobacco use (29%) in the past 12 months. Sixteen percent reported history of depression, and 14% history of anxiety. On average, participants scored 1.4 (SD 1.5) on the mental health/substance use index. Sixty-nine percent had previously given birth, and 28% had previously had an abortion.

**Table 1:** Description of sample (N = 586).

Variable	n (%) or mean ± SD
State	
Louisiana	282 (48)
Maryland	304 (52)
Age, in years (M, SD)	27.0 **±** 5.6
Race/ethnicity	
Black or African American	461 (79)
Hispanic/Latina	55 (9)
White	45 (8)
Other/Multiple	24 (4)
Highest level of education	
Less than high school	120 (20)
Completed high school or GED	286 (49)
Some or completed college	179 (31)
Employment	
Employed full time	176 (30)
Employed part time	122 (21)
Unemployed	285 (49)
Insurance status	
Uninsured	88 (15)
Employment/Self/Other	58 (10)
Medicaid	432 (75)
Public assistance in last 12 months	431 (75)
Housing insecurity in last 12 months	172 (30)
Food insecurity in last 12 months	271 (47)
Any binge drinking	205 (36)
Alcohol use disorder risk	153 (26)
Any drug use	112 (19)
Tobacco use	164 (29)
Any depression	93 (16)
Any anxiety	78 (14)
Previous CPS involvement	56 (10)
Previous birth	401 (69)
Previous abortion	165 (28)
Economic insecurity index (M, SD)	2.1 ± 1.2
Mental health/substance use index (M, SD)	1.4 ± 1.5

Thirty-one percent (n = 182, 77/282 in Louisiana and 105/301 in Maryland) reported that they had considered abortion during their pregnancy. Three percent (n = 19) reported a policy-related barrier as a reason they did not have an abortion, with policy-related barriers higher in Louisiana (5%, or 15/278) than in Maryland (1%, or 4/301). Overall, 11% (19/181) who considered abortion reported a policy-related barrier; this was 20% (15/76) in Louisiana and 4% (4/105) in Maryland.

#### Factors associated with not having an abortion

In bivariate analyses, state of residence, race/ethnicity, previous birth, previous abortion, employment, insurance status, public assistance, housing insecurity, food insecurity, depression, anxiety, binge drinking, alcohol use disorder risk, drug use, and tobacco use were associated with having considered abortion. Both indices were associated with having considered abortion.

In regression analyses using individual economic status and mental health/substance use variables, being uninsured (aOR 0.69 95% CI [0.50, 0.95]) was associated with lower odds and food insecurity (aOR 1.68 95% CI [1.16, 2.42]), drug use (aOR 1.36 95% CI [1.08, 1.70]), and anxiety (aOR 1.72 95% CI [1.19, 2.47]) were associated with higher odds of having considered abortion. Hispanic/Latina ethnicity was associated with lower odds and previous birth and previous abortion were associated with higher odds of having considered abortion. All other variables were not associated with having considered abortion (Table 2).

**Table 2:** Predictors of having considered abortion and having faced a policy barrier to abortion.

	Considered abortion		Faced a policy barrier to abortion	
	Model 1 aOR (95% CI) n = 554	Model 2 aOR (95% CI) n = 583	Model 1 aOR (95% CI) n = 566	Model 2 aOR (95% CI) n = 579
State				
Maryland (ref.)	--	--	--	--
Louisiana	1.04 (0.74–1.46)	0.93 (0.66–1.31)	**5.80 (4.04–8.32)**	**6.06 (4.52–8.13)**
Race/ethnicity				
African American (ref.)	--	--		--
White	0.70 (0.44–1.13)	0.64 (0.33–1.24)	0.33 (0.06–1.86)	0.26 (0.05–1.33)
Hispanic/Latina	**0.56 (0.33–0.96)**	**0.55 (0.31–0.99)**	0.37 (0.13–1.04)	0.33 (0.10–1.08)
Other/Multi	0.32 (0.05–2.25)	0.36 (0.05–2.68)	0.77 (0.06–9.07)	0.73 (0.08–6.68)
Previous birth	**2.06 (1.40–3.02)**	**1.80 (1.38–2.35)**	**4.05 (2.09–7.84)**	**3.99 (1.80–8.82)**
Previous abortion	**1.70 (1.21–2.38)**	**1.70 (1.29–2.23)**		
Unemployed	1.16 (0.95–1.43)			
Uninsured	**0.69 (0.50–0.95)**			
Public assistance	0.85 (0.70–1.02)		1.35 (0.83–2.20)	
Housing insecure	1.25 (0.94–1.65)		1.24 (0.32–4.90)	
Food insecure	**1.68 (1.16–2.42)**		2.86 (0.76–10.76)	
Alcohol use disorder risk	1.70 (0.87–3.32)			
Any drug use	**1.36 (1.08–1.70)**			
Tobacco use	0.77 (0.54–1.09)		**2.50 (1.91–3.29)**	
Depression	1.52 (0.94–2.46)			
Anxiety	**1.72 (1.19–2.47)**			
Economic insecurity index		**1.21 (1.17–1.26)**		**1.50 (1.09–2.08)**
Mental health/substance use index		**1.29 (1.16–1.45)**		**1.45 (1.03–2.05)**

p < .05 in bold.

Model 1 includes all variables found to be significantly associated with outcome in bivariate analysis (p < .10), with the exception of any binge drinking due to its high correlation with alcohol use disorder risk. Model 2 replaces mental health and substance abuse dichotomous variables with a continuous index. All standard errors clustered by recruitment site.

In regression analyses using indices, with exception of state of residence, all factors associated with having considered abortion in bivariate analyses were associated with having considered abortion. Women with greater economic insecurity (aOR 1.21 [95% CI 1.17, 1.26]) and more mental health diagnoses/substance use (aOR 1.29 [1.16, 1.45] had higher odds of having considered abortion. Again, Hispanic/Latina ethnicity was associated with lower odds, while previous birth and previous abortion were associated with higher odds of having considered abortion.

#### Factors associated with reporting a policy-related barrier

In bivariate analyses, state of residence, race/ethnicity, previous birth, public assistance, housing and food insecurity, and tobacco use were associated with reporting a policy-related barrier as a reason. Both indices were associated with reporting a policy-related barrier. In regression analyses with individual economic security and individual mental health diagnosis and substance use items, only state of residence, tobacco use, and previous birth were associated with reporting policy-related barriers to care (Table 2).

In regression analyses with indices, greater economic insecurity (aOR 1.50 [1.09, 2.08]) and more mental health diagnoses/substance use (aOR 1.45 [1.03, 2.05]) had higher odds of reporting a policy-related barrier. In addition, women living in Louisiana and women who had a previous birth had higher odds of reporting a policy-related barrier.

In regression analyses restricted to the sample of those who had considered abortion, findings were similar to those in the overall sample in terms of direction of effects and point estimates, although no longer statistically significant due to reduced sample size.

In regression analyses restricted to the Louisiana sample with individual economic insecurity and individual mental health diagnosis and substance use items, being uninsured (aOR 0.70 95% CI [0.53, 0.92] was associated with lower odds of reporting a policy-related barrier while food insecurity (aOR 1.96 95% CI [1.50, 2.54]), alcohol use disorder risk (aOR 5.78 95% CI [3.80, 8.79]), and tobacco use (aOR 1.47 95% CI [1.02, 2.12]) were associated with higher odds of reporting a policy-related barrier. Previous birth was also associated with higher odds of reporting a policy-related barrier (Table 3).

**Table 3:** Predictors of having faced a policy barrier to abortion among Louisiana participants.

	Faced a policy barrier to abortion	
	Model 1 aOR (95% CI) n = 272	Model 2 aOR (95% CI) n = 278
Race/ethnicity		
African American (ref.)	--	--
White	0.43 (0.07–2.48)	0.23 (0.04–1.23)
Hispanic/Latina	0.60 (0.16–2.20)	0.38 (0.11–1.27)
Other/Multi	1.29 (0.04–41.69)	0.89 (0.05–16.89)
Previous birth	**5.00 (1.55–16.15)**	**3.28 (1.56–6.90)**
Uninsured	**0.70 (0.53–0.92)**	
Public assistance	0.98 (0.57–1.69)	
Food insecure	**1.96 (1.50–2.54)**	
Alcohol use disorder risk	**5.78 (3.80–8.79)**	
Any drug use	2.09 (0.57–7.70)	
Tobacco use	**1.47 (1.02–2.12)**	
Economic insecurity index		1.39 (0.95–2.04)
Mental health/substance use index		**1.66 (1.37–2.00)**

p < .05 in bold

Model 1 includes all variables found to be significantly associated with outcome in bivariate analysis (p < .10), with the exception of any binge drinking due to its high correlation with alcohol use disorder risk. Model 2 replaces mental health and substance abuse dichotomous variables with a continuous index. All standard errors clustered by recruitment site.

In regression analyses with indices, greater economic insecurity was not associated with reporting a policy-related barrier (aOR 1.39 [95% CI 0.95, 2.04]). Having more mental health diagnoses and substance use was associated with higher odds of reporting a policy-related barrier (aOR 1.66 [95% CI 1.37, 2.00]). Previous birth was also associated with higher odds of reporting a policy-related barrier.

### Qualitative findings

#### In-depth interview sample description

Of the forty-three women recruited in Southern Louisiana who completed an interview, twenty-eight discussed having considered an abortion for this pregnancy in their interview. These twenty-eight women ranged in age from 18 to 38, with most in their 20s. Twenty-three identified as Black, three as Hispanic, and two as White. Most (n = 17) were already parenting. Fourteen had completed high school or less and thirteen had completed some college. Twelve were employed, three were full-time students, and the remaining thirteen were unemployed. At the time of the interview, one respondent was deciding between placing the baby for adoption and parenting; the others all planned to continue their pregnancies and parent. Seven interviewees who, during the in-clinic interview, identified personal and/or interpersonal reasons as a reason they did not obtain an abortion, described facing policy-related barriers to abortion in their in-depth interviews.

#### Policy barrier: Prohibition on public funding for abortion

Louisiana’s restriction on public funding for abortion means that people seeking an abortion must pay out-of-pocket for the abortion. Whether that cost represents a hardship depends on the personal circumstances of the person considering abortion. For the women interviewed, all low-income or poor, it was almost universally a hardship. Fourteen respondents described the out-of-pocket cost of abortion as something they could not afford. For these respondents, the challenges of having to pay for abortion out-of-pocket were complex.

As Vanessa explained, “I found out I was pregnant and then I wasn’t going to keep it at first. But then once [I realized] I didn’t have the money for it, I was like, I have to keep it now.” Vanessa had been diagnosed with depression, bipolar disorder, and anxiety and reported in her in-clinic survey that she occasionally used drugs. She had a history of suicidal ideation. When she discovered her pregnancy, she tried to raise the money from friends and family, calling around and asking people for money. She “wasn’t able to get anything” from her efforts to fundraise. Ultimately, Vanessa considered it very hard to get an abortion in Louisiana because, as she said, “you need money to do it. You have to pay for it.” Already barely making ends meet and navigating mental health issues, the out-of-pocket cost of abortion was too high for her. Although Vanessa considered and wanted an abortion, as soon as the clinic told her the cost over the phone, she demurred from even scheduling an appointment, understanding the cost of abortion to be beyond her reach.

Paige, who reported drinking ten or more drinks at a time two to three times a week and occasional drug use in her in-clinic survey, likewise wanted an abortion when she discovered she was pregnant and called a clinic to schedule an appointment. The clinic staffer explained the pricing system and Paige realized an abortion was beyond her means. She did not have the money. She asked her boyfriend for money and he had nothing to offer. She estimated that, were she to try “extra, extra, extra hard,” she might be able to raise about $200, which would fall short of the amount she needed to pay for an abortion. As she summed up, “[the money] was part of the reason why I couldn’t, why I didn’t do it [the abortion]”.

Like both Vanessa and Paige, several respondents asked others for money to pay for their abortion. They were typically met with refusals, as the men involved in the pregnancy as well as their family and friends expressed opposition to abortion. Shaunice, who reported using drugs weekly in her in-clinic survey, asked both her husband and her mother for money to pay for an abortion. Both refused out of their ideological opposition to abortion. Shaunice’s ability to obtain an abortion was squarely premised on access to funds. She explained how impactful the funding was in describing her request for a loan from her mother: “if she gave me the money, I would do it.” In Shaunice’s case, like several others, opposition to abortion by others had consequential power in whether these women obtained an abortion, a power it would not have were the costs of abortion covered by insurance.

#### Policy barrier: Gestational limits

The time it took for some respondents to raise money to pay for their abortion made them subject to a second policy-related barrier to abortion: gestational limits. Jayla, for example, did not have the money to pay for the abortion she wanted. She reported binge drinking on a weekly basis and daily drug use in her in-clinic survey as well as a housing situation where she considered her activities not to be good for the pregnancy. She was already 10 weeks pregnant at the time she discovered her pregnancy. Such late recognition of pregnancy is common for people, like Jayla, who drink more heavily and use drugs frequently [17, 18]. She asked her mother for financial help. Although her mother was initially reluctant, asking her to “think about it,” she eventually agreed to give Jayla the money for the abortion. It took Jayla’s mother a little while to gather the money and “when my mom finally got enough money for the abortion, that’s when my mom called them, and they told my mom that [there] was the cutoff.” Although, as Jayla explained, “we all knew that there was a deadline,” she had not realized it was so close. By the time she had the money from her mother, Jayla realized she was beyond the facility’s gestational limit and would have to continue the pregnancy. As Jayla’s experience demonstrates, the challenge represented by having to pay for abortion out-of-pocket did not exist in a vacuum and often was connected to other state and institutional policies.

#### Policy barrier: Two-visit requirement

Other policy-related factors interacted with lack of Medicaid funding to make an abortion unfeasible for respondents. Tyler, for example, had the out-of-pocket costs for a first trimester procedure, but by the time her work schedule allowed her to take enough time off to accommodate the state-mandated two-visit requirement, she was in the second trimester and unable to afford the out-of-pocket costs for a second trimester procedure. For Tyler, who reported drinking three or four drinks at a time two to three times a week in her in-clinic survey, the most significant impediment to her obtaining an abortion was the two-visit requirement: “they said it was going to be like a three-day process, and I really didn't have three days to give them”.

Maria anticipated that the cost of an abortion would be high and felt overwhelmed by the prospect of having to find the money. At the same time, she was unable to treat her diagnosed depression because she ran out of medication and was waiting to see a new psychiatrist for a new prescription. Obtaining an abortion in Louisiana was hard, she said, because “Medicaid won’t pay for the abortion.” As she tried to save funds, she put off calling a clinic for an abortion. By the time she called a clinic, she was told by one that she was over their facility limit. The other clinic she contacted was willing to see her, and explained she would have to have two separate visits and state-mandated counseling. Maria wanted a timely abortion and was put off both by the required counseling and the fact that she would have to return twice. She explained, “I thought it was just a process you say, you know, you want it done and you go in and you’re going to have it done. But no, it was a process that you have to go, you have to be checked and then they have to check you.” After spending weeks raising the money for the abortion and considering her decision, that extra time was unappealing to Maria: “I wasn’t trying to wait any longer.” She gave up on pursuing abortion and resigned herself to continuing her pregnancy.

#### Knowledge of abortion policies

Comprehensive knowledge of what an abortion entailed logistically was rare, only one respondent reported knowing about the two-visit requirement before deciding to seek an abortion. Based on a friend’s experience, Madison, who reported drinking three or four drinks at a time two to three times a week and weekly drug use in her in-clinic survey, knew she would have to make two visits to the clinic. Because of that foreknowledge, she scheduled an abortion counseling appointment soon after she began considering abortion, albeit before she had decided whether abortion was indeed the right decision for her. Nonetheless, even with her preparation, Madison was surprised to learn that the clinic she visited could not accommodate her medical circumstances and referred her instead to a clinic several hours drive away. When she called that clinic, she learned that they would not guarantee they could care for her until she came for an appointment. Still deciding whether abortion was the right decision for her, the difficulty of getting to a clinic that could care for her—and having to go twice, to fulfill the two-visit requirement—figured into her decision-making and she eventually decided, “I’m not going to go out of my way to do something [complete abortion counseling] that I’m not even [sure I am] wanting to do anyhow”.

## Discussion

Drawing on survey and structured interview data, this study found that self-reported both greater economic insecurity and more mental health diagnoses and substance use are associated with having considered but then not having an abortion and with reporting a policy-related barrier as a reason for not having an abortion. In the more restrictive policy environment, only more mental health diagnoses and substance use as reported by the participants were associated with policy-related barriers. Drawing on in-depth interviews, it also found that women in Louisiana identified policies–such as those that ban use of state funds to pay for low-income women’s abortions, those that contribute to gestational limits on available abortion care, and those that require two visits to an abortion clinic to be able to obtain an abortion–as part of what made it difficult for them to obtain abortion care. Importantly, these policies do not appear to operate in isolation. Rather they, in some cases, interact with each other (as in the case of lack of public funding delaying people past gestational limits) and potentially function as difficult if not impossible to surmount barriers to abortion care for women with individual complex situations. These individual complex situations may be further compounded by anti-abortion attitudes of family members and partners who do not want to contribute material support to help women obtain abortions.

We note that, in analyses restricted to Louisiana, greater economic insecurity was not associated with reporting a policy-related barrier to abortion. The point estimate is similar to the overall analyses (1.50 vs. 1.39) and the p-value is 0.09, just above traditional cutoffs for statistical significance; thus, the lack of statistical significance in the Louisiana sample is likely due to reduced power. Thus, both our quantitative and qualitative findings are consistent with other research that finds that economic insecurity is a barrier to abortion care [31, 32]. Previous qualitative research indicates that drug use is a barrier to prenatal care [33] and previous research indicates that substance use–especially heavier use–can contribute to later discovery of pregnancy [17, 18] and substance use and mental health can contribute to delays in presenting for abortion care [19]. This study indicates, though, that these factors do not just contribute to delays in presenting for care, but may contribute to some women being unable to obtain an abortion at all. Restrictive abortion policies may thus be more likely to be insurmountable for women with complex individual situations–situations with economic insecurity and mental health and substance use.

Another key contribution that differentiates experiences of economically insecure women with complex situations considering abortion care from those considering and seeking prenatal care is that abortion care was often stigmatized in their communities. Interviewees described how lack of Medicaid funding for abortion care and prohibitions on when in pregnancy abortion care is available, as compared to prenatal care, was consequential and how opposition to abortion from family members and partners impeded their ability to overcome these policy barriers: not only did some women lack funds to pay for abortion, for example, but they reported that people in their lives refused to help them *because* the money was for abortion.

A strength of our mixed-methods approach is that we confirmed that more women experience a policy-related barrier to abortion than previous research that focused only on the quantitative aspects of this study suggest. In in-depth interviews, multiple women who had not cited a policy-related barrier as a reason they did not have an abortion in the in-clinic quantitative component gave accounts that included barriers to their having an abortion that were rooted in restrictive policies, primarily related to lack of Medicaid funding for abortion care. This supports our earlier contention that the quantitative policy-related barrier results are likely an undercount of policy-related reasons for not having an abortion [5]. Another strength of our mixed-methods approach is the identification of anti-abortion attitudes of family members as contributing to women’s difficulties overcoming policy-related barriers to care. From a methodological perspective, both of these findings suggest the in-depth interview method allows people to reference multiple factors that influence their experience, while, the quantitative portion is, as would be expected, less expansive. Based on the qualitative analyses alone, though, we may have missed the key role that mental health and substance use play in not being able to overcome policy-related barriers as the interview guide did not include domains on these topics. While some in-depth interview participants mentioned mental health or substance use, it was not discussed in-depth in all interviews. Another strength of the study is the high participation rate (86%) across both states.

There are a number of limitations. First, the sample reporting a policy-related barrier as a reason they did not have an abortion in the quantitative component is small (n = 19), which only allows detection of large effects and also makes any point estimate imprecise. Second, our measure of policy-related barriers is imprecise. In terms of implications for findings, we observed mostly similar patterns for considering abortion, which suggests patterns of findings in terms of which women are more likely to experience a policy-related barrier are likely accurate. Third, our economic insecurity and mental health/substance use indices are not validated measures. They also count each component equally, when one aspect may have more bearing than others. Relatedly, as noted above, the interview guide did not include domains on mental health/substance use, reducing the ability of the transcripts to offer insight into how these factors mattered for respondents. Fourth, while the convergent and embedded mixed methods data collections enabled us to collect data via multiple modes in a limited time frame, thereby reducing retrospective bias, it meant we could not design the qualitative mode to examine particular findings of the quantitative mode. Fifth, the study was conducted with primarily low-income women and women of color recruited at four university-affiliated prenatal care sites in regions of two states. Thus, findings may not be generalizable to higher income women, White women, or women living in other states or regions of states we studied.

There are a few implications. First, this study suggests current restrictive abortion policies have more impact on women with greater economic insecurity and women with more mental health diagnoses and substance use. This means that these groups are disproportionately impacted by restrictive abortion policies; they are also groups who may need additional support to be able to obtain abortions and, when not able to obtain abortions, to obtain services during their pregnancy and afterwards. In fact, our previous research has found that women who report policy-related barriers to abortion report needing services such as substance use disorder treatment [34]. Previous research underscores the importance of thinking about how to support women unable to obtain abortions, as this research indicates that denying women abortion does not resolve factors (such as problematic alcohol use or economic insecurity) that existed prior to her becoming pregnant or that led her to consider abortion in the first place [13, 35].

## Conclusions

Women who report that they are economically insecure and who have more mental health diagnoses/substance use appear more affected by current restrictive abortion policies. These policies appear to interact with each other and with people’s individual complex situations, including their economic, mental health, and substance use, as well as with anti-abortion attitudes of other people to become barriers to abortion care.

## Supporting information

S1 File In-clinic survey and interview questions. (PDF)

S2 File In-depth Interview Guide. (DOCX)
