# Supplementary material for: Complex situations: Economic insecurity, mental health, and substance use among pregnant women who consider – but do not have – abortions
Source: PLoS One. 2020 Jan 15;15(1):e0226004. doi: 10.1371/journal.pone.0226004 (PMC6961826; doi:10.1371/journal.pone.0226004)
Supplement: S1 File — (PDF) [file pone.0226004.s001.pdf]

STUDY ID: ________________________

Self-Administered Survey

(Questions used in analyses presented in Roberts SCM, Berglas NF, Kimport K. Complex situations: economic insecurity, mental health, and substance use among pregnant women who consider – but do not have – abortions.)

The following questions should be answered by filling out this form. The research coordinator will leave you alone to answer the questions. She is available to answer any questions you may have while you are taking the survey. She will let you know where to find her. Once you have completed the self-administered survey, please find the research coordinator. **Your answers will not be shared with the research coordinator or your health care provider. Please answer all questions.**

<u>Questions About You</u>

The first questions ask about your background.

1. What is your date of birth? _________________ (MM/DD/YYYY)

2. What state do you live in now? _______

3. What is the highest grade or year of school you have completed?
   - ☐ Eighth grade or less
   - ☐ Some high school
   - ☐ High school graduate or GED
   - ☐ Associates degree
   - ☐ Some college or technical school
   - ☐ College graduate
   - ☐ Post graduate or professional degree

4. How would you describe your work status now?
   - ☐ Employed full time
   - ☐ Employed part time
   - ☐ Unemployed
   - ☐ Homemaker
   - ☐ Unable to work
   - ☐ Other (please specify) ___________________________

5. Are you Hispanic or Latina?
   - ☐ Yes
   - ☐ No

6. Which one of these groups would you say best represents your race?
   - ☐ White
   - ☐ Black or African American
   - ☐ Asian
   - ☐ Native Hawaiian or Other Pacific Islander
   - ☐ American Indian or Alaska Native
   - ☐ Other (Specify Race) _____________________________
   - ☐ Two or more races/multiracial (Specify Races)________________________

The next series of questions have to do with abortion. We know that this is a sensitive subject for some people, and it can be difficult to talk about. We want to remind you that the goal of our study is to understand women's experiences with pregnancy and healthcare, whatever they are. We know that when women learn that they are pregnant, they often consider several different options. Sometimes, these options include abortion. We have no preferences or judgments about abortion, adoption, or parenting. **Your answers will <u>not</u> be shared with your doctor or nurse or with the research coordinator who conducts your interview.**

When women find out they are pregnant, they have three options: having the baby and raising it, adoption or having someone else raise the baby, or having an abortion. These next questions are about your decision-making for this pregnancy.

7. Have you considered having an abortion for this pregnancy even for just one second?
   - ☐ Yes
   - ☐ No

<u>Questions about other pregnancies</u>

8. Before this pregnancy, how many times in your life have you been pregnant? Please include live births, miscarriages, abortions, and stillbirths? ___________ (If zero, skip to question 12)

9. How many children have you given birth to? _____________

10. How many miscarriages or stillbirths have you had? _____

11. As a parent, have you ever been involved with Child Protective Services?
    - ☐ No
    - ☐ Yes

    26a. If you answered yes, which of the following best describes your involvement with Child Protective Services?
    - ☐ CPS removed one or more of my children permanently
    - ☐ CPS removed one or more of my children temporarily
    - ☐ CPS investigated me, but never removed any of my children
    - ☐ Other: ____

STUDY ID: ________________________

<u>**Questions about your health**</u>

Remember, your answers will <u>not</u> be shared with your doctor or nurse or with the research assistant who conducts your interview.

12. Has a doctor or other health professional (for example: social worker, therapist, psychiatrist) ever told you that you had any of the following mental health conditions? (Check all that apply)
    - ☐ A depressive disorder like major depression, depression, dysthymia, or bipolar disorder
    - ☐ An anxiety disorder like panic, obsessive-compulsive, anxiety, or post-traumatic stress disorder
    - ☐ Any other mental health condition we haven't mentioned (Specify)__________ _______________
    - ☐ None of the above

Thinking about the 12 months <u>before you found out you were pregnant</u>, please answer the following questions:

13. In the 12 months before you found out you were pregnant, how often did you have a drink containing alcohol?
    - ☐ Never (skip to question 16)
    - ☐ Monthly or less
    - ☐ 2 to 4 times a month
    - ☐ 2 to 3 times a week
    - ☐ 4 or more times a week

14. One drink is equivalent to a 12-ounce beer, a 5-ounce glass of wine, or a drink with one shot of liquor. In the 12 months before you found out you were pregnant, how many drinks containing alcohol did you have on a typical day when you were drinking?
    - ☐ 1 or 2
    - ☐ 3 or 4
    - ☐ 5 or 6
    - ☐ 7, 8, or 9
    - ☐ 10 or more

15. In the 12 months before you found out you were pregnant, how often did you have 4 or more drinks on one occasion?
    - ☐ Never
    - ☐ Less than monthly
    - ☐ Monthly
    - ☐ Weekly
    - ☐ Daily or almost daily

STUDY ID: ________________________

16. In the 12 months before you found out you were pregnant, how often did you use any illicit or street drugs or prescription drugs for recreational use?
    - ☐ Never
    - ☐ Less than monthly
    - ☐ Monthly
    - ☐ Weekly
    - ☐ Daily or almost daily

17. How many cigarettes or packs of cigarettes did you smoke on an average day? (A pack has 20 cigarettes) *Please choose one*
    - ☐ [# of] _________cigarettes
    - ☐ [# of] _________packs
    - ☐ Less than one cigarette a day
    - ☐ I did not smoke

<u>Questions about your financial situation</u>

18. Do you currently have any kind of health care coverage or insurance? Include Medicaid, LaMOMS, and LaHIPP
    - ☐ Yes
    - ☐ No (if no, skip to question 20)

19. Do you receive health care coverage or insurance through: (Check all that apply)
    - ☐ Your job
    - ☐ Someone else's job
    - ☐ A plan that you or someone else pays for out of pocket, like private insurance or Obamacare
    - ☐ Medicaid that you received specifically for this pregnancy, such as Medicaid or LaMOMS
    - ☐ Medicaid that you had before this pregnancy, such as Medicaid, LaMOMs, LaHIPP
    - ☐ Some other source (Specify **Source**): ___________________________

20. During the last 12 months, was there a time when you or your family were <u>not</u> able to pay your mortgage, rent, or utility bills?
    - ☐ Yes
    - ☐ No

The following are statements that people have made about their food situation. Mark whether the statement was often true, sometimes true, or never true during the last 12 months. If you live alone, answer just for yourself. If you live with your family, please answer for your family.

21. I/we worried whether my/our food would run out before I/we got money to buy more.
    - ☐ Often true
    - ☐ Sometimes true
    - ☐ Never true

22. The food that I/we bought just didn't last, and I/we didn't have money to get any more.
    - ☐ Often true
    - ☐ Sometimes true
    - ☐ Never true

23. In the last 12 months, did you/you and other adults in your family ever cut the size of your meals or skip meals because there wasn't enough money for food?
    - ☐ Yes
    - ☐ No

24. During the past 12 months, have you received any money or assistance from the government? (Check all that apply)
    - ☐ Temporary Assistance to Needy Families (TANF), welfare
    - ☐ WIC
    - ☐ Food Stamps
    - ☐ Social Security/Disability
    - ☐ Medicaid, LaMOMs, or LaHIPP
    - ☐ Other (Please specify): ____________
    - ☐ I did not receive any money or assistance from the government

STUDY ID: ________________________

In-Clinic Interview (Administered by the Research Coordinator)

(Questions used in analyses presented in Roberts SCM, Berglas NF, Kimport K. Complex situations: economic insecurity, mental health, and substance use among pregnant women who consider – but do not have – abortions.)

Thank you again for taking the time to participate in this study. I want to remind you that the information you share will be kept confidential. We will not share your answers with your health care provider. Remember, you do not have to answer any questions that you do not feel comfortable answering. But your answers are very important to us and will help us understand women's health care needs in Louisiana. This part involves both multiple choice and some questions where I'm going to ask you to tell me in your own words.

Many pregnant women consider abortion, even for just one second, during their pregnancies. The next questions focus on your experiences considering or seeking abortion during this pregnancy. I know that this can be somewhat difficult to talk about. I want to reassure you that the information you share with me is confidential. I will not share any of what you tell me with your doctors or nurses.

1. Did you consider having an abortion during this pregnancy even for one second?
   - ☐ Yes
   - ☐ No (skip questions 2 - 16)

I'm going to read you a series of steps that women who consider abortion may take after considering abortion. For each step, I'd like you to tell me whether you took that step or did not take that step during this pregnancy.

2. Did you call a clinic to find out about having an abortion during this pregnancy? Please answer yes to this question even if you tried to call a clinic, but did not actually get through to a person at a clinic.
   - ☐ Yes (skip to question 5)
   - ☐ No

3. What are the reasons you did not call a clinic to find out about having an abortion during this pregnancy? (Probe with Anything else? Until she stops offering a response.)

4. What would you say is the main reason you did not call a clinic to make an appointment? (skip to question 16)

5. Did you make an appointment for an abortion during this pregnancy?
   - ☐ Yes (skip to 8)
   - ☐ No

6. What are the reasons you did not make an appointment for an abortion during this pregnancy? (Probe with Anything else? Until she stops offering a response.)

7. What would you say is the main reason you did not make an appointment?
   (skip to question 16)

8. Did you go to an abortion counseling/information visit during this pregnancy? By abortion counseling/information visit, we mean a visit to clinic that provides abortions where doctors, nurse, or other staff gave you information about what would be involved in having an abortion.
   - ☐ Yes (skip to question 11)
   - ☐ No

9. What are the reasons you did not go to an abortion counseling/information visit during this pregnancy? (Probe with Anything else? Until she stops offering a response.)

10. What would you say is the main reason you did not go to an abortion counseling/information visit during this pregnancy? (skip to question to 16)

11. Did you go to an abortion appointment during this pregnancy? By appointment, we mean a visit to have the abortion.
    - ☐ Yes
    - ☐ No (skip to question 14)

12. What are the reasons you did not have the abortion after going to the abortion appointment? (Probe with Anything else? Until she stops offering a response.)

13. What would you say is the main reason you did not have the abortion after going to the abortion appointment? (skip to question 16)

14. What are the reasons you did not go to the abortion appointment? (Probe with Anything else? Until she stops offering a response.)

15. What would you say is the main reason you did not go to the abortion appointment?

16. Thinking about all of the factors that have made it so you have not had an abortion during this pregnancy, what would you say is the main reason you have not had an abortion during this pregnancy?
