# Supplementary material for: Complex situations: Economic insecurity, mental health, and substance use among pregnant women who consider – but do not have – abortions
Source: PLoS One. 2020 Jan 15;15(1):e0226004. doi: 10.1371/journal.pone.0226004 (PMC6961826; doi:10.1371/journal.pone.0226004)
Supplement: S2 File — (DOCX) [file pone.0226004.s002.docx]

**Abortion Prenatal Study In-depth Interview Guide**

**Background & Demographics**

- 1. What is your age?
  2. How do you identify your race or ethnicity?
  3. Are you currently working? If yes, what do you do? If no, if you were working, what would you like to be doing?
  4. Current state and city of residence
  5. How many people are in your household? What are their ages and relationships to you?
  6. Do you have any children who do not live with you?
  7. Are you currently pregnant? If yes, how many weeks gestation?

**Pregnancy Experience - Current**

1. Can you tell me about how you came to be at [recruitment site] for your prenatal appointment? How did you learn about it? Have you been there before?
2. What was your experience there? Who did you see? What happened? Did anyone come with you?
   1. What went well during this visit? What, if anything, went badly during this visit?
   2. Did anything surprise you about this visit?
   3. Was there anything you needed that you didn’t get?
   4. Have you been back since?
   5. **For women who started prenatal care after the first trimester**: did the doctor or nurse ask you about your reasons for starting prenatal care later in pregnancy? If so, could you tell me about that conversation?
3. How did you first learn you were pregnant?
4. What were your first thoughts and feelings when you discovered you were pregnant?
5. Who did you tell that you were pregnant first? Can you tell me about that conversation?
6. Who else have you told since? What reactions have you gotten? Has anyone reacted negatively?
7. Do you know/remember when you got pregnant?
8. Who was the man? Can you tell me about your relationship with him?
9. Did you go to a pregnancy resource center? If yes:
   1. How did you learn of the center? Had you been before?
   2. Why did you decide to go? What were you expecting would happen at your visit?
   3. Can you walk me through your (first) visit (for this pregnancy) there?
   4. How did you feel about your visit?
   5. Did you learn anything at your visit? If yes, what?
   6. Did your visit make you feel or think differently about your pregnancy? If yes, how?
   7. Would you recommend going to the center to a friend who was pregnant? Why or why not?
10. What are your plans for this pregnancy? [If she is no longer pregnant, ask this instead: can you tell me about how your pregnancy ended?] Has/had that [parenting, adoption, having someone else raise the child—fill in with language used by respondent] always been your plan? If no:
    1. What other plans did you have for this pregnancy? How certain were you of that earlier plan?
    2. What did you do to make that happen? What got in your way of making that happen?
    3. Did you tell other people about your plan? If yes, who? How did they react?
    4. **For women who considered abortion or had an abortion**, have you talked with any doctors or nurses about having considered abortion for this pregnancy? If yes, can you tell me about that conversation?
    5. **For those who considered abortion and are now planning to parent/place child for adoption,** what are the reasons you decided not to have an abortion? Can you walk me through that decision.
    6. **For women who have had an abortion,** where did you have the abortion?
       1. Was there anything that got in the way of having an abortion? If yes, how did you overcome it?
       2. Was there anything that especially made it possible for you to have an abortion?
11. How do you feel about your pregnancy right now? [For women who are no longer pregnant, ask instead, How do you feel about that pregnancy now?]

**Pregnancy Experience - Previous**

- - - 1. Have you been pregnant before? If yes, how many times?
      2. For each prior pregnancy: How old were you? What were the circumstances of conception? What was the outcome of that pregnancy?
         1. For any parenting/adoption, did you consider any other option, like parenting/adoption or abortion? Why did you choose [parenting/adoption]?
    1. For any abortions, Can you tell me about that experience? How did you decide to have an abortion?

**Knowledge of and Positions on Abortion**

1. Do you know anyone [else] who has had an abortion? How did you come to know about their abortion(s)?
2. What kind of women do you think get abortions?
   1. Are there women who should get abortions? Women who shouldn’t get abortions?
3. What would you say to someone [a friend] who told you she was pregnant and thinking about getting an abortion?
4. Do you know about some of the recent laws around abortion in Louisiana? Probe for what she knows and sources.
   1. Why do you think government made these laws?
   2. Did any of the laws affect your decision for this pregnancy?
   3. Do you think any of the laws affected your ability to have an abortion for this pregnancy?
5. Do you think it’s easy or hard to get an abortion in Louisiana? Is that a good thing?
6. Can you imagine a scenario where you would want/need an abortion? If yes, can you tell me what you’re imagining?

**The goal of this project is to understand women’s experiences of pregnancy in Louisiana. Are there any other experiences, feelings, or thoughts on this topic that you think would help my understanding?**
